# Pattern and trends of the total and age-specific fertility rates during 1990–2018 in Pakistan

**DOI:** 10.1186/s12905-023-02435-8

**Published:** 2023-06-06

**Authors:** Lubna Naz, Asifa Kamal, Adan Kamran, Kassahun Trueha

**Affiliations:** 1 Department of Economics, Institute of Business Administration, Karachi, 75270 Pakistan; 2 Department of Statistics, Lahore College for Women University, Lahore, Pakistan; 3 Department of Statistics, Wolkite University, Welkite, Ethiopia

**Keywords:** Age-specific fertility rate, Direct methods, Relational Gompertz model, Total fertility rate

## Abstract

**Background:**

Pakistan has an inadequate vital event registration system, leading to fewer than half of all births being registered, and this issue is further exacerbated by systematic recall errors and omission of births. This study aims to evaluate direct and indirect methods of fertility estimation to analyze the trends and patterns of fertility rates in Pakistan from 1990 to 2018.

**Design/methodology/approach:**

Indirect methods are utilized in this study to evaluate the direction and extent of changes in total and age-specific fertility rates, and these findings are compared to direct estimates. The study draws data on livebirths from four waves of the Pakistan Demographic and Health Survey that took place between 1990 and 2018. To ensure the quality of data, graphical methods and Whipple and Myers indices are employed. Additionally, the Brass Relational Gompertz model was used to analyze the data.

**Results:**

The Relational Gompertz model revealed that total fertility rates (TFRs) were higher than direct estimates by 0.4 children and age-specific fertility rates (ASFR) were higher for all age groups except the oldest. The difference was more significant among younger women aged 15–24, and less so for age groups 29 and above. The gap in estimated fertility between direct and indirect methods decreased with age.

**Conclusion:**

The indirect method is an invaluable tool in situations where direct measurement of fertility rates is challenging or impossible. By utilizing this method, policymakers can gain important insights into the fertility patterns and trends of a population, which is crucial for making informed decisions on fertility planning.

**Supplementary Information:**

The online version contains supplementary material available at 10.1186/s12905-023-02435-8.

## Introduction

Pakistan lacks a complete registration system of vital events, resulting in over half of under the age of five (58%) being unregistered (National Institute of Population Studies, 2017–18) [1]. The major reasons for inadequate vital event registration include a lack of knowledge about birth registration among parents, insufficient resources for birth registration, and births in remote areas [1–4].

The Pakistan Demographic and Health Survey (PDHS) serves as the primary source for examining current and lifetime fertility. It collects birth history data in temporal sequence from all women of child-bearing age (15–49 years) [5]. The data is used to compute the direct estimates of fertility indices such as the “age-specific fertility rate (ASFR)”, “total fertility rate (TFR)”, and “crude birth rate (CBR)”. Moultrie et al. [6] classified methods for estimating fertility into three categories, one of which is direct estimation of fertility.

The direct estimation of fertility can be done in three possible ways depending on the availability of data. In the first approach to direct estimation of fertility, the vital registration system is used. The second method uses full birth history data collected in the survey, and the third approach employs summary fertility measures collected routinely in censuses for the estimation of recent fertility. Direct estimates of fertility are helpful to assess the changes in the pattern of fertility [7].

In the indirect estimation of fertility, information on the lifetime fertility of younger women collected through the census or survey is used in conjunction with direct estimates of fertility to adjust them. However, response errors are common in birth history data [8]. The errors are typically in the form of recall errors in reporting age and birth events and omissions of births [9, 10].

Apart from that, systematic recall errors are also a big issue [11]. For example, uneducated mothers (or both parents) usually do not maintain records of the date of births of their children. Hence, when asked to report the birth date of a child or the total number of births, it is highly likely that they would either underreport or overreport. The errors caused by underreporting or overreporting distort birth estimates and generate biased results of fertility decline [10]. Researchers usually impute birth data for the missing values in household surveys to make up for the recall errors. However, in the presence of many missing observations, the imputation procedure may significantly affect the quality of the fertility estimates derived from surveys [5, 8].

The impact of these intrinsic errors can be reduced by using indirect methods of fertility estimations [7]. The Relational Gompertz model is used to provide indirect estimate of fertility. Indirect methods allow for a review and analysis of the past and present data on fertility and the examination of changes in fertility rates [7, 9]. However, the choice of appropriate indirect methods is subject to the assumptions related to the nature of data and estimation technique. For example, some indirect methods are based on inappropriate assumptions for countries experiencing rapid demographic transition, and may therefore produce biased estimates [8, 12, 13].

The Relational Gompertz Model is commonly used as an indirect method to determine the shape and level of fertility in a population while accounting for irregularities such as age misreporting of children and births in the data [6].

In Pakistan, the total fertility rate has shown a declining trend over the years. From 1970 to 1980, the rate was between six to seven children per woman. [14], and in the1980s, it was estimated to be at 6.5 per woman [3]. Official figures suggest a decline in TFR from 7.1 children in 1960 to 6.3 children per woman in 1975 [15]. This decline can be attributed to the increase in the age at first marriage [14]. However, a previous study using “own children method” provided evidence against the declining fertility rate, suggesting that the inception of fertility decline was an artefact of the data [16].

The total fertility rate in Pakistan was reported to be more than six children in 1990–91 (PDHS, 1990–91). However, a further fall was observed in the following years, with a TFR of 4.1 in 2006–07 and 3.8 in 2012–13. Between 2012–13 and 20,171–18, there was a negligible decrease of 5% in TFR (National Institute of Population Studies, 2017–18) [1]. These estimates of TFR were computed using a direct method and reported in all Pakistan Demographic and Health Surveys or PDHS, which are known to be prone to errors and unreliable.

Previous studies have projected that Pakistan would achieve the replacement-level of fertility by 2020 [4]. To support the projections, the data on the average number of children ever born for all women showed a decline from 3.0 in 1990–91 to 2.13 2017–18. However, this contrasts with the tremendous increase in the population reported by the “Sixth Housing and Population Census of Pakistan 2017”, raising concerns about the use of direct methods to estimate fertility [17]. Pakistan’s population increased from 129 million in 1989 to 207 million in 2017, moving from fourteenth to the fifth position among the world’s most populous countries [17].

Therefore, it can be assumed that fertility decline trends reported by previous studies based on direct methods were not precise. Limited literature exists on the implications of using the direct versus indirect methods for estimating total and age-specific fertility rates in Pakistan [2, 4, 14, 16, 18, 19]. However, the available evidence is either restricted to single method or outdated and reassessing the pattern and trends of total and age-specific fertility is necessary.

This study fills the research gap by examining the pattern and trends of the total and age-specific fertility rates (from 1990 to 2018) with the help of indirect methods. It uses the Brass P/F ratio and Relational Gompertz model, which accounts for errors and omissions in births. Further, it compares fertility estimates obtained by direct methods with those derived from indirect methods. The study uses four waves of Pakistan Demographic and Health Survey covering the period of 28 years. The research findings help to explain the fertility puzzle in Pakistan and adds to the empirical evidence on fertility transitions in a developing country, which is crucial for devising population control strategies and programs.

## Methods and material

### Data and sample

This study used four waves of Pakistan Demographic and Health Surveys: (PDHS, 1990–91, PDHS 2006–07 PDHS 2012–13, and PDHS 2017–18). Sample size comprised of 6611, 10,023, 13,557 and 15,068 women respectively for each survey wave. We have used the PDHS for the computation of TFR and considered the women of reproductive age group 15–49 year. Our analysis is restricted to the mean number of children born to a biological mother. Therefore, the children co habiting with the family at the time of survey were not the part of the analysis. DHS collects data on birth histories from all eligible married women of 15–49 years of households randomly selected for the interview.

The surveys were implemented by the National Institute of Population Studies in close coordination with the Ministry of National Health Services, Regulations, and Coordination Surveillance. Surveys followed a two-stage stratified random design. Firstly, regions were stratified into urban and rural. Secondly, the primary sampling units (PSUs) were selected using the information on the enumeration blocks and following a probability rule, probability proportional to size or PPS, from the sampling frame developed by the Pakistan Bureau of Statistics.

Finally, secondary sampling units (SSU) or households were selected through systematic random sampling from the selected Enumeration Blocks (EBs) of urban areas. In rural areas, households were chosen from mouzas/dehs/villages. This study mainly used data from birth histories in each survey. Total fertility rates used were from the 36 months prior to each survey. Similarly, age-specific fertility rates were computed for births occurring within the previous three years of each survey.

### Relational Gompertz model

This study is divided into two parts: data evaluation and fertility estimation using indirect methods of estimation. Data evaluation is necessary to strengthen our confidence in the reliability of estimates obtained. To avoid documenting extensive details of the data evaluation procedures and results in the paper, a supplementary file has been prepared and attached to the manuscript.

The study used the refined form of the Brass P/F technique, Known as the Relational Gompertz model (RGM), to estimate the level and trend of fertility [20–22]. Brass P/F was used as a benchmark measure of fertility because it is a useful diagnostic tool for analyzing the errors present in the fertility data. The Brass P/F ratio method relies on the following assumption that if fertility remains unchanged over time, the period and long-term fertility rates tend to be the same [12].

The RGM serves as the best-suited diagnostic tool to identify data errors. If Brass P/F departs from a hypothesized value of “1”, it implies some errors or omissions in the fertility data. Two types of errors occur in the data if the value of Brass P/F deviates from “1”. First, if women underreport recent births, irrespective of their age, it inflates the P/F value. Second, if older women underreport their lifetime fertility, it decreases the numerator, and consequently, P/F ratio deflates [8, 12].

The RGM estimates ASFRs and TFRs by defining the fertility schedule form with the help of the data on birth histories and shaping the pattern from reported average parities of younger women [23]. The fertility estimates produced by the RGM are reliable because it automatically corrects common errors observed in fertility data [20].

The cumulative function of the Gompertz distribution has a sigmoid shape (S-shaped), representing the average parities of women through age. Moreover, the hazard function of Gompertz distribution is right skewed, capturing the pattern of cumulated fertility of women [20]. The Gompertz distribution function $G\left(x\right)$ is linearized for age by taking a double negative log transformation, as follows:

1 $$Y\left(x\right)=-ln\left(-ln\left(G\left(x\right)\right)\right)$$

Equation (1) refers to gompit. Brass found that gompitz of observed series of fertility data closely fits as a straight-line function with the gompit of a defined standard fertility schedule.

2 $$Y\left(x\right)=\alpha +\beta {Y}^{S}\left(x\right)$$

In Eq. (2), ${Y}^{S}\left(x\right)$ is the gompit of standard fertility schedule and $Y\left(x\right)$ is gompit of observed fertility schedule. If $\alpha =0$ and $\beta =1$, the observed and standard fertility schedule are identical. The value of α represents the extent to which age location of childbearing women in the population differs from that of the standard, and a negative sign (-) with α indicates that the women have a relatively higher age distribution of childbearing compared to the standard. $\beta$ quantifies the spread of fertility distribution, with a value of $\beta >1$ indicating a narrower distribution [20, 24]. This method has two disadvantages. First, TFRs computed through age-specific fertility rates are used as inputs, which may be biased. Second, there is an embedded assumption of constant fertility over time.

This study utilized the Microsoft Excel worksheet for data analysis, specifically the “Relational Gompertz model” (RGM) as outlined by [6]. Direct methods were used to obtain the fertility estimates, which were subsequently recalculated using tfr2 commands in STATA (version 14) following the approach outlined by previous studies [25].

## Results

Table 1 displays the decreasing trend in total fertility over time for the three countries; computation were based on using direct and indirect method. Findings suggest that India is approaching towards replacement-level of fertility. Total fertility rates obtained by Relational Gompertz model are higher compared to those derived using direct method for each country. Furthermore, the decline in total fertility is more pronounced for India, followed by Bangladesh and then Pakistan.

**Table 1:** Comparison of TFRs using direct method and relational Gompertz model for Pakistan, India and Bangladesh

Country	Survey	TFR	
		**Direct**	**Indirect (Relational Gompertz model)**
**Pakistan**	PDHS 1990–91	5.4	5.8
	PDHS 2006–07	4.1	4.8
	PDHS 2012–13	3.8	4.4
	PDHS 2017–18	3.6	4.0
**India**	NFHS1992-93	3.4	4.2
	NFHS 1998–99	2.8	3.9
	NFHS 2005–06	2.7	3.5
	NFHS 2015–16	2.2	2.7
**Bangladesh**	BDHS 1993–94	3.4	5.1
	BDHS 1996–97	3.3	4.6
	BDHS 1999–00	3.3	4.2
	BDHS 2004	3.0	4.0
	BDHS 2007	2.7	3.6
	BDHS 2011	2.3	3.2
	BDHS 2014	2.3	3.1

Pakistan’s highest rate of decline in fertility was -1.82 during 2012 2017 (Table 2). in contrast, the annual percentage decline in fertility from 1990 to 2017 was “-1.15”, indicating a 1.15% decrease in fertility rate per year since 1990. For India, the annual percentage decline in fertility from 1992 to 2015was “-1.55”, suggesting a 1.55% decrease in fertility rate per year since 1992. Bangladesh had a “-1.87” annual percentage decline in fertility from 1993 to 2014, showing the highest decline in fertility amongst the three countries, and perhaps in the region as well.

**Table 2:** Total Fertility Trends in Pakistan, India, and Bangladesh

Country	Survey	%Decline		Annual % Decline
		**Direct**	**Indirect**	
**Pakistan**	PDHS 1990–91 to PDHS 2006–07	-24.1	-17.2	-1.08
	PDHS 2006–07 to PDHS 2012–13	-7.3	-8.3	-1.39
	PDHS 2012–13 to PDHS 2017–18	-5.3	-9.1	-1.82
	PDHS 1990–91 to PDHS 2017–18	-33.3	-31.0	-1.15
**India**	NFHS1992-93 to IDHS 1998–99	-17.6	-7.1	-1.19
	NFHS 1998–99 to IDHS 2005–06	-3.6	-10.3	-1.47
	NFHS 2005–06 to IDHS 2015–16	-18.5	-22.9	-2.29
	NFHS 1992–93 to IDHS 2015–16	-35.3	-35.7	-1.55
**Bangladesh**	BDHS 1993–94 to BDHS 1996–97	-2.9	-9.8	-3.27
	BDHS 1996–97 to BDHS 1999–00	0.0	-8.7	-2.90
	BDHS 1999–00 to BDHS 2004	-9.1	-4.8	-0.95
	BDHS 2004 to BDHS 2007	-10.0	-10.0	-3.33
	BDHS 2007 to BDHS 2011	-14.8	-11.1	-2.78
	BDHS 2011 to BDHS 2014	0.0	-3.1	-1.04
	BDHS 1993 to BDHS 2014	-32.4	-39.2	-1.87

This study computed Brass P/F ratios for all four waves of Pakistan Demographic and Health Survey: “PDHS 1990–91, PDHS 2006–07, PDHS 2012–13, and PDHS 2017–18,” covering a period of almost 28 years (Table 1). Age-specific-fertility rates (ASFR) were computed for the 36 months preceding each survey and were used as input for both the Brass P/F and Relational Gompertz model estimations. This study observed variations in in P/F ratios (Table 3) across different age groups, with the differences being more pronounced among younger women (age 20–29). P/F ratios for age groups, ranging between 20–24 and 25–29, were less than one (< 1), indicating a higher current fertility. A similar pattern of P/F ratio was observed across all waves of PDHS. The pattern could be due to underreporting of lifetime fertility or overreporting of current fertility.

**Table 3:** The brass P/F ratio, age-specific fertility rates, parities, and total fertility rates in Pakistan from 1990–91 to 2017–18

Age	PDHS 1990–91					PDHS 2006–07				
	Direct		Indirect		P/F ratio	Direct		Indirect		P/F ratio
	MCEB 1991	ASFR 1991	ASFRs (true age)	Parities		MCEB 2007	ASFR 2007	ASFRs (true age)	Parities	
10–14	-	-	0.002	0.001	-	-	-	-	-	-
15–19	0.2	0.084	0.124	0.21	-	0.08	0.051	0.078	0.093	-
20–24	1	0.23	0.274	1.288	0.640	0.72	0.178	0.243	0.954	0.572
25–29	2.6	0.268	0.279	2.709	0.849	2.14	0.237	0.26	2.272	0.815
30–34	4.3	0.229	0.23	3.995	0.998	3.77	0.182	0.201	3.444	1.018
35–39	5.5	0.147	0.162	4.98	1.057	4.97	0.106	0.126	4.265	1.129
40–44	6.3	0.073	0.072	5.575	1.102	5.57	0.044	0.046	4.693	1.174
45–49	6.4	0.04	0.009	5.745	1.096	6.31	0.018	0.004	4.79	1.312
Total	3	5.4	5.75	-	-	2.53	4.1	4.79	-	-
Age	PDHS 2012–13					PDHS 2017–18				
	Direct		Indirect		P/F ratio	Direct		Indirect		P/F ratio
	MCEB 2013	ASFR 2013	ASFRs (true age)	Parities		MCEB 2018	ASFR 2018	ASFRs (true age)	Parities	
10–14	-	-	-	-	-	-	-	-	-	-
15–19	0.06	0.044	0.068	0.075	-	0.07	0.046	0.061	0.066	-
20–24	0.69	0.19	0.229	0.868	0.53	0.65	0.171	0.216	0.794	0.529
25–29	1.99	0.224	0.244	2.115	0.726	1.91	0.215	0.245	2.008	0.76
30–34	3.51	0.181	0.185	3.207	0.93	3.22	0.16	0.195	3.132	0.939
35–39	4.5	0.091	0.111	3.947	1.029	4	0.079	0.124	3.936	1.011
40–44	5.29	0.03	0.038	4.314	1.143	4.92	0.028	0.046	4.359	1.175
45–49	5.98	0.007	0.003	4.392	1.282	5.31	0.012	0.004	4.455	1.258
Total	2.42	3.8	4.4	-	-	2.13	3.55	4.459	-	-

-MCEB is Mean Children Ever Born

The increase in fertility at younger age groups implies younger women might have overreported their current fertility. Moreover, the discrepancies in reporting women’s age may cause misplacement of reported births [7, 11, 18]. Age heaping, differential coverage by age, and errors in reporting childbirth date were found during data quality check that might have affected the P/F ratio (see Supplementary file).

The P/F ratio is not a direct measure of fertility, but rather a tool used to measure fertility based on reported births. It is also subject to various sources of error, such as underreporting or overreporting of births, misreporting of women's age, and discrepancies in reporting of births by month and year. Despite these limitations, the P/F ratio is still considered a useful tool for estimating fertility, particularly in the absence of reliable birth registration systems or other direct measures of fertility [4, 13, 25, 26].

The P/F ratio for women aged 30–39 is considered adequate if it approximates “1”, as it indicates that reporting errors in current and lifetime fertility are cancelled out [12]. Across all years, P/F ratio increases with women’s age (Table 3). As fertility is not static in Pakistan, the P/F ratio exceeding one (P/F > 1) suggest a decline in fertility. Moreover, the pattern of P/F ratio increment is consistent for all age groups and years, except for women aged 45–49 in 1990–91.

A higher P/F ratio observed for the age group 45–49 compared to younger age groups suggest the possibility of age misreporting among older women. This could lead to an underestimation of the number of children ever born in this age group. Additionally, older women may have difficulty accurately recalling the number of children they have had due to memory lapses. Another factor that may contribute to reporting errors is the omission of deceased or out-of-home children in the 1990–91 survey. However, despite these potential sources of error, the overall trend in P/F ratios across all age groups and survey years suggests a decline in fertility, which was more pronounced in the 2006–07 survey compared to the 1990–91 survey.

### Comparison between direct and indirect estimates of fertility

**Fig. 1:**
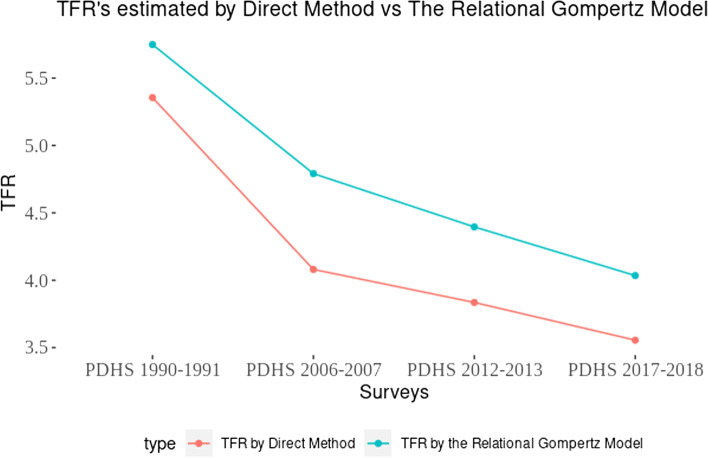
Trends in total fertility rates by direct method vs indirect method from 1990–91 to 2017–18

The estimates of TFRs using RGM for all surveys were higher than the TFRs derived using direct methods (Fig. 1).

**Fig. 2:**
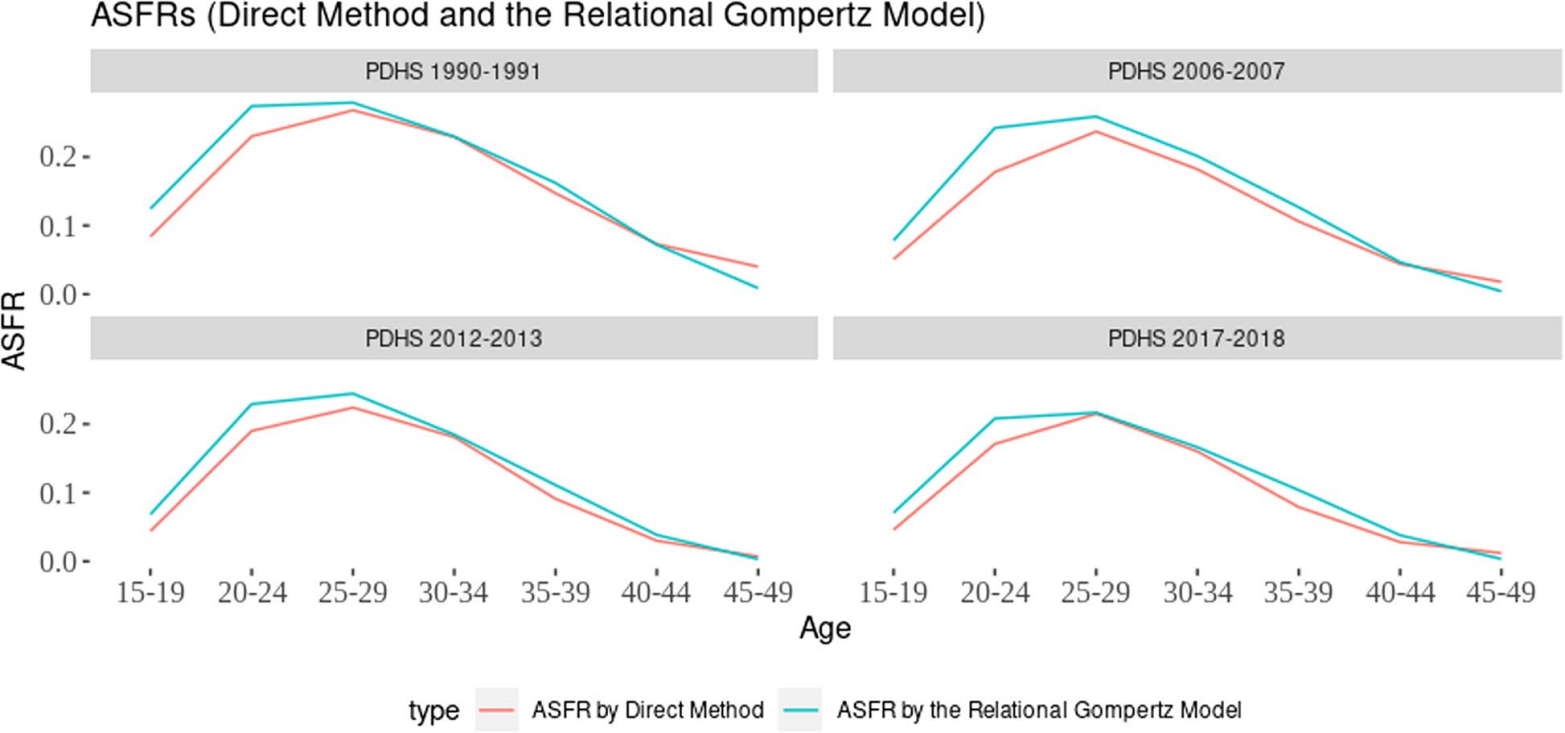
Age specific fertility rates by direct method and Relational Gompertz model from 1990–91 to 2017–18

The indirect method (Fig. 2) revealed that the observed fertility (ASFR) calculated from direct estimates was underestimated. The RGM automatically adjusted for errors and produced higher estimated fertility than direct fertility estimates in all survey years, except for older age groups 40–49. The difference between direct and indirect estimates was most pronounced among younger age groups (29 years and below). As age increased, the gap in estimated fertility between direct and indirect methods narrowed down. The maximum difference was observed in the age group 20–24. However, after age 40, the indirect estimate of fertility became lower than the direct estimate. This pattern was observed in all survey years, except in 2012–13. The results indicate that both direct and indirect methods produced similar fertility estimates for age groups (20–24 and 40 and above).

### Fertility pattern and trend in Pakistan: relational Gompertz model

**Fig. 3:**
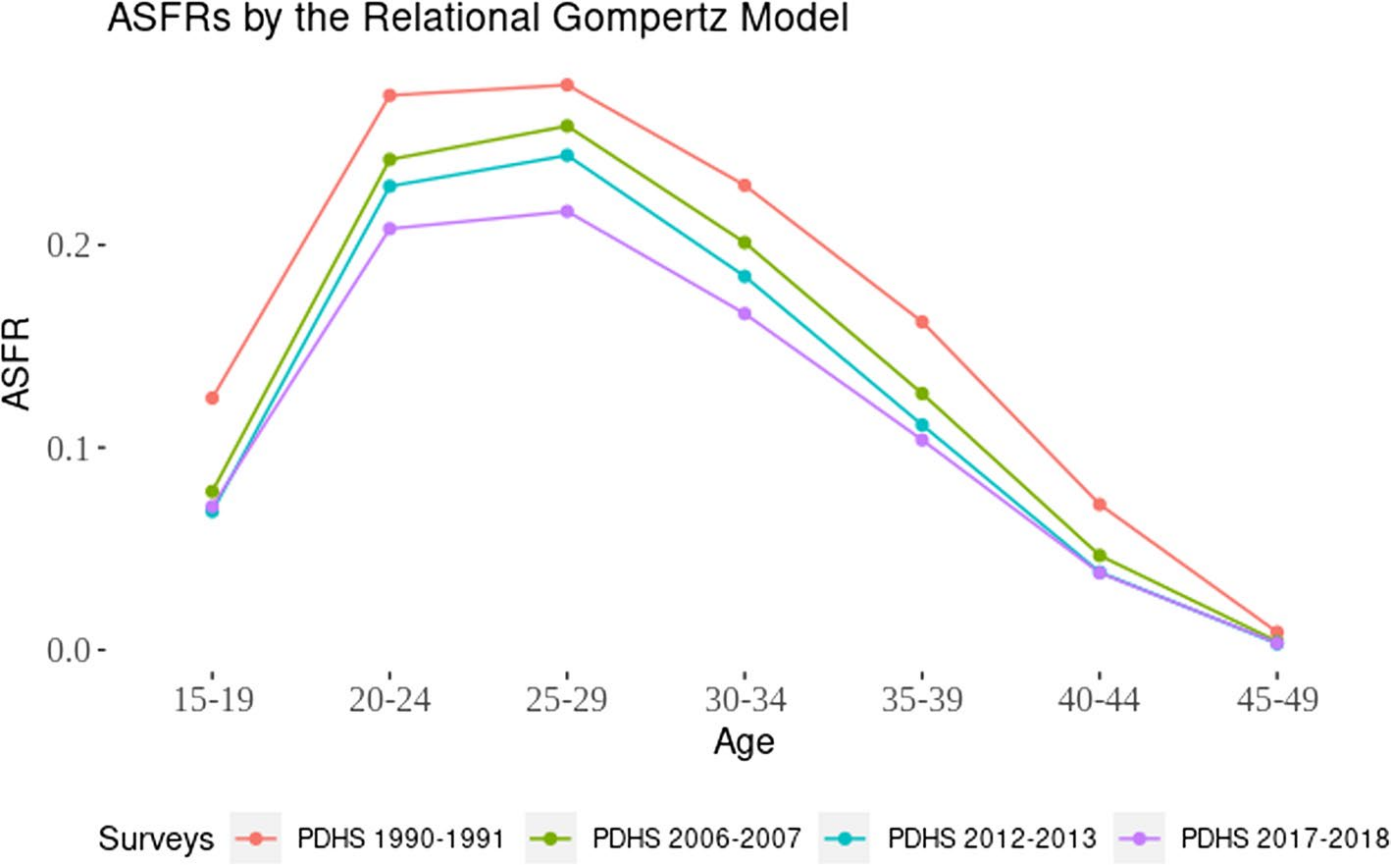
Age specific fertility rates by Relational Gompertz model from 1990–91 to 2017–18

Figure 3 depicts a consistent and gradual decline in fertility over time. The reduction in ASFR was most significant from the earliest to the most recent survey year. However, the decline in ASFRs in 2012–13 and 2017–18 was negligible among younger age groups (15–24 years) and older age groups (35–49 years), while substantial decline was observed among middle-aged age groups (25–34 years). The fertility rate was highest among women aged 20–29, with most women achieving their desired number of children by age 39. After age 29, fertility started declining, and this decline tended to become more pronounced as woman’s age increased.

**Fig. 4:**
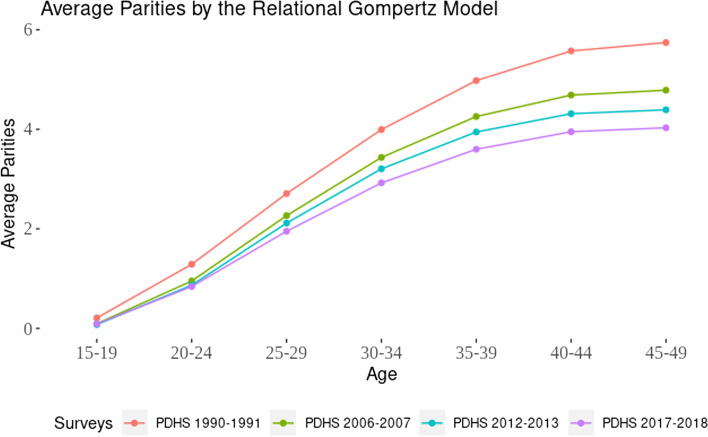
Average parities by Relational Gompertz model from 1990–91 to 2017–18

Figure 4 shows a consistent decline in average parities over 28 years from 1990–91 to 2017–18. The decline in average parities was steady for younger age groups across all survey years, while for older age groups, the decline was more prominent in earlier and later years. The difference in average parities varied depending on the time interval between surveys, with wider intervals resulting in more pronounced differences, indicating a consistent decline in fertility at a slower pace. The corrected age parities suggest that childbearing in Pakistan at the youngest age declined substantially after 1990–91, but no further significant reduction was observed in later years.

The parameter estimates computed from RGM for all surveys were within the range shown in Tables 4 and 5. α represents the extent to which childbearing women of various age groups deviate from the standard. The value of α was negative but very small for all surveys, which implies a slightly older distribution of childbearing ages (beyond age 27) compared to the standard, Table 5.

**Table 4:** The trend of the total fertility decline by direct and indirect methods (1990–2018)

Survey	TFR		Survey	% Decline		Annual % Decline
	Direct	Indirect		Direct	Indirect	
PDHS 1990–91	5.4	5.8	1990–91 to 2006–07	-24.1	-17.2	-1.08
PDHS 2006–07	4.1	4.8	2006–07 to 2012–13	-7.3	-8.3	-1.39
PDHS 2012–13	3.8	4.4	2012–13 to 2017–18	-5.3	-9.1	-1.82
PDHS 2017–18	3.6	4.0	1990–91 to 2017–18	-33.3	-31.0	-1.15

**Table 5:** The values of Alpha and Betas for various PDHS

Surveys	α	$\beta$
PDHS 1990–91	-0.082	1.030
PDHS 2006–07	-0.120	1.153
PDHS 2012–13	-0.111	1.195
PDHS 2017–18	-0.089	1.155

The distribution of childbearing ages among women in Pakistan has been changing over time, see Table 4. While around half of the women who gave birth were aged 27 in both the earliest and latest survey years, a slightly older distribution of age (beyond age 27) was observed during the period from 2006 to 2012. However, the value of α decreased in the most recent survey year (2017–18), suggesting a shift in childbearing age closer to 27. However, significant improvements could not be made due to the negligible increase in the proportion of educated women over the years. Furthermore, lack of awareness has led to poor maintenance of birth history records even by educated mothers at home.

The parameter β, which measures the spread of the fertility distribution, was greater than one in all survey years, indicating a narrower distribution. Over time, the distribution of fertility has become thinner and was the narrowest in the most recent survey year (2017–18), see Tables 4 and 5.

The reasons for slow fertility decline were traced by comparing changes in proximate factors (Table 6) and selected women’s background characteristics (Table 7).

**Table 6:** Changes in proximate determinants of fertility (1990–91 to 2017–18)

Survey	Median Age at first Marriage Women 15–49	Median Age at First Birth Women 15–49	Median Breastfeeding Duration (months)	Median Birth interval (months)	Median Duration of Amenorrhoea (months)	Median Duration of Abstaining (months)	Median Duration of Insusceptible (months)	Current use of Contraceptive currently married Women 15–49	Mean Ideal Number of Children women
PDHS 1990–91	18.6	21.3	20	29.1	6.3	2.4	7.5	11.8%	4.1
PDHS 2006–07	19.1	21.8	18.3	28.8	3.9	2.1	4.8	29.6%	4.1
PDHS 2012–13	19.5	22.2	18.3	28	3.6	2	4.4	35.4%	4.1
PDHS 2017–18	20.4	22.8	18	28.2	3.3	2.4	4	34.2%	3.9

**Table 7:** Changes in women background characteristics (1990–91 to 2017–18)

Survey	Education		Work Status	Residence Urban Women
			**Currently employed**	
	**No**	**Higher**		
**PDHS 1990–91**	79.20%	1.10%	16.80%	30.50%
**PDHS 2006–07**	65.00%	6.40%	25.90%	33.40%
**PDHS 2012–13**	57.10%	9.30%	26.30%	33.50%
**PDHS 2017–18**	49.50%	10.30%	17.30%	36.80%

Both the median ages at first marriage and first birth increased by 1.8 and 1.5 years respectively between 1990–91 and 2017–18 in Pakistan. This can be considered as one of the factors contributing to the consistent decline in fertility rates in the country.

## Discussion

Previous studies have established a consistent decline in the total fertility rate in Pakistan, particularly after 1990. However, the evidence was based on direct estimates of fertility that are prone to errors. The unusual pattern in the birth distribution was identified and reported in PDHS 2012–13. Age displacement and omission of children were the probable reasons for these discrepancies [27]. Dead children were underreported in PDHS 2012–13. Male dead children were also underreported in PDHS 1990–91 [28].

This study provides estimates of fertility patterns and trends in Pakistan by accounting for data errors. The Relational Gompertz model (RGM) was used instead of the Brass P/F ratio method, which makes assumptions about constant fertility that may not hold in developing countries with incomplete or distorted data [29]. Similar studies preferred RGM to overcome limitations of Brass P/F method in the estimation of fertility trends [5, 7].

The total fertility estimates obtained by RGM showed a decline over the years in Pakistan. According to RGM estimates, the TFR was four children per woman. The decline in total fertility rate was estimated to be 31% between 1990–91 and 2017–18. The estimates of TFR obtained by RGM were found to be consistently higher in all survey years compared to the fertility rates estimated by direct methods. Recent surveys such as the Pakistan Demographic Survey (PDS, 2020) and the Pakistan Social and Living Standards Measurement (PSLM, 2018–19) reported TFR as 3.7 which is slightly higher than the 3.6 reported in PDHS 2017–18, supporting the argument that direct estimates marginally underestimate actual fertility rates [30, 31].

A difference of at least 0.4 children was observed between the TFRs for direct and indirect estimations. The overall annual rate of decline in TFR for 28 years depicted by the indirect method was 1.15% (Table 2). From 1990–91 to 2006–07 (17 years), the annual average decline in fertility was approximately 1.1% per year. The annual rate of decline increased by 13.9% during 2006/07–2012/13, whereas the highest decline (1.82%) was recorded between 2012–13 and 2017–18. However, the decrease in TFR presented by indirect estimates was lower than direct estimates. A recent study observed a difference in the estimates of fertility provided by direct and indirect method of fertility estimation. Under estimation was observed in the direct fertility estimates at both urban/rural and at regional level [32].

This study found the absolute value of α increased in the middle two survey years (2006–07 and 2012–13) but again declined during 2017–18 (Table 5). As α became negative, it indicates that childbearing age slightly shifted to older ages. This suggests that the age at first birth would have increased in the middle two survey years compared to the earliest one (1990–91). A similar trend was observed for β. The value of β kept increasing until 2012–13, and then slowed down although the trend of narrowness decreased in 2017–18. Increased women’s education, increased female labour force participation, decreased fertility intention, and increase in the modern contraceptive prevalence may have played significant roles in bringing down fertility [3].

On the other hand, a two-month decline was observed in the median duration of breastfeeding among childbearing mothers. The median birth interval shortened by one month between 1990–91 and 2012–18, whereas it increased by one and a half months in 2017–18 [1]. Similarly, a decrease was also noticed in the median duration of amenorrhea and insusceptibility. These developments in maternal healthcare utilization may have slowed down the total fertility rate. Moreover, the data revealed an increase in the use of modern contraceptives over time. However, it has been established that contraceptives were used for birth spacing rather than terminating births. The woman stops childbearing after attaining her or her husband’s fertility intentions. Reportedly, the fertility intention of women has remained static in Pakistan at 4.1 children per woman.

Based on the findings presented, there are several policy implications that can be drawn for fertility planning in Pakistan, as follows. 1) the findings indicate regional disparities in fertility rates, with higher rates observed in rural areas and in some provinces such as Balochistan and Khyber Pakhtunkhwa. Family planning programs should be targeted to address these regional disparities, 2) the fertility intentions of women have remained static at 4.1 children per woman, indicating the need to address underlying factors that influence fertility intentions, such as gender norms, education, and economic empowerment of women, 3) results call for investing into maternal and child health factors, such as breastfeeding duration and birth intervals, to influence fertility rates. Programs aimed at improving maternal and child health can contribute to reducing fertility rates in Pakistan, 4) accurate age-specific fertility estimation can help policymakers identify which age groups have the highest fertility rates, and subsequently design targeted interventions to reduce fertility rates in these age groups.

## Conclusion

The indirect method offers reliable estimate of fertility in countries where misreporting about childbirth and women’s age are common. It may be due to a massive illiteracy and inadequate vital event management system. This can help policymakers to better understand the fertility patterns of a population and make informed decisions about resource allocation and family planning programs.

However, the accuracy of indirect methods may be limited by the assumptions underlying the model and the quality of the data used to calibrate it. Therefore, policymakers should be cautious in interpreting and using the results of indirect methods and consider them in conjunction with other sources of data and information. In addition to this, the evidence provided by this study may not be applicable to other populations or contexts. Further research would be needed to confirm these findings and explore the underlying causes and potential policy implications in similar settings.

## Supplementary Information

**Additional file 1: Supplementary material. **Data quality checks.

## Data Availability

This article is based on the secondary data set. The following is the link of Pakistan Demographic Health Surveys used in this manuscript. https://dhsprogram.com/data/available-datasets.cfm. The data is in open domain. Anyone can access the data and replicate the results with prior permission of the DHS program-ICF.
