# Supplementary material for: Pattern and trends of the total and age-specific fertility rates during 1990–2018 in Pakistan
Source: BMC Womens Health. 2023 Jun 6;23:300. doi: 10.1186/s12905-023-02435-8 (PMC10245426; doi:10.1186/s12905-023-02435-8)
Supplement: Supplementary file 1 — Additional file 1: Supplementary material. Data quality checks. [file 12905_2023_2435_MOESM1_ESM.pdf]

Supplementary Material: Data Quality Checks

Estimation of error is beneficial in assessing the degree of confidence in estimates computed even though error is not corrected (Preston et al. 2001). Halbana (2006) also argued that care should be used in application of methods for correcting these errors as it affects integrity of data.

1. Assessment of Parity Data

1.1 Implausible Parities

There was no implausible parity found in the PDHS 2017-18 and PDHS 2006-07. One implausible parity was found in PDHS 2012-13 and one in PDHS 1990-91 for age group 25-29 reporting 13 and 16 children respectively.

1.2 Assessment of Enumerator Errors

No blanks for parity are found in fertility data collected in all PDHS Surveys. Hence, there was no need to apply el-Badry correction.

1.3 Proportion of Women Childless

Proportion of childlessness decreased in all PDHS surveys with age group of women. Proportion of childlessness in oldest age group (45-49) was 0.2%, 0.3%, 0.4% and 0.3% for PDHS 2017-18, PDHS 2012-13, PDHS, 2006-07 and PDHS 1990-91 respectively. Problem for further investigation was not required as childlessness was less than 10% for oldest age group (Figure 1).

Figure 1: Percentage of Childlessness

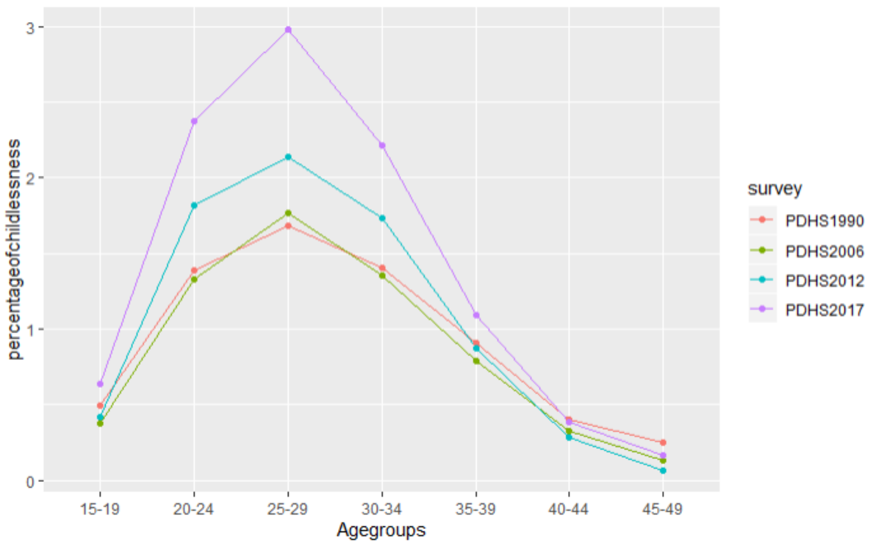

1.4 Average Parities

Shape of all curves for mean children ever born mean number of living children across age of mother were sigmoid. Rising trend was also observed so distortion in average parities were not suspected.

Figure 2: Average Parities

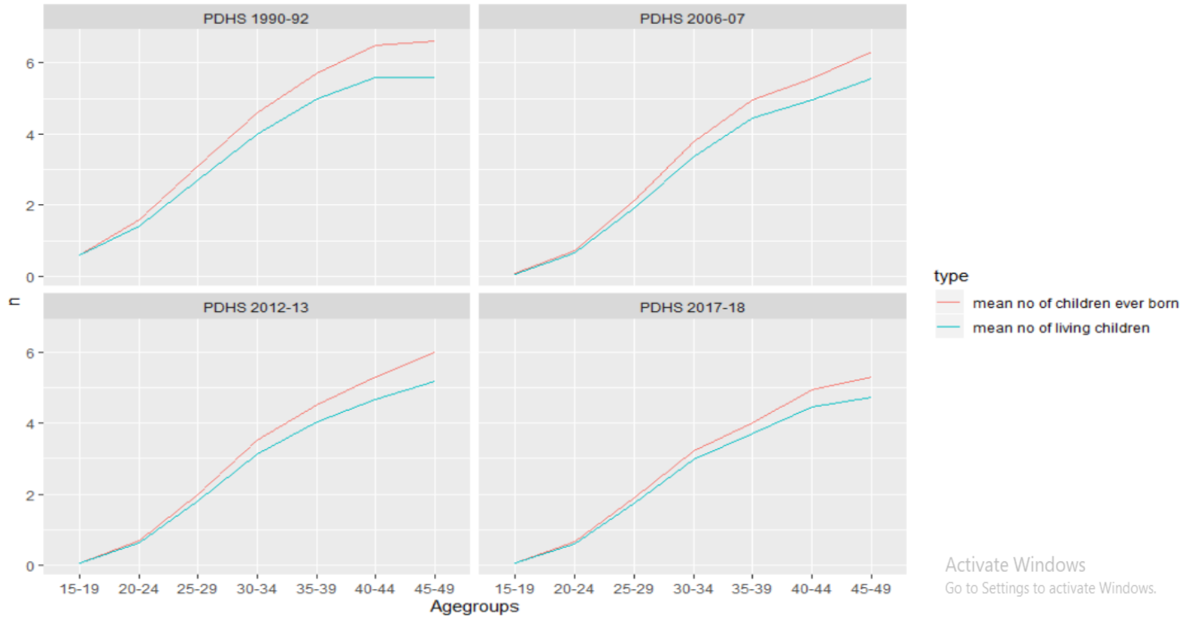

1.5 Comparison with Total Fertility

For all surveys parity of older women was greater than TFR as expected if fertility is declining (Table1). If older age group of women under report children ever born, then average parity will be depressed. This under reporting also decreases TFR artificially due to omissions of birth in recent fertility.

Table1: Average Parity and TFRs

Survey	Average Parity (45-49)	TFR
**PDHS 1990-91**	6.40	5.4
**PDHS 2006-07**	6.30	4.1
**PDHS 2012-13**	5.98	3.8
**PDHS 2017-18**	5.31	3.6

Figure3: Average Parities by Age Group

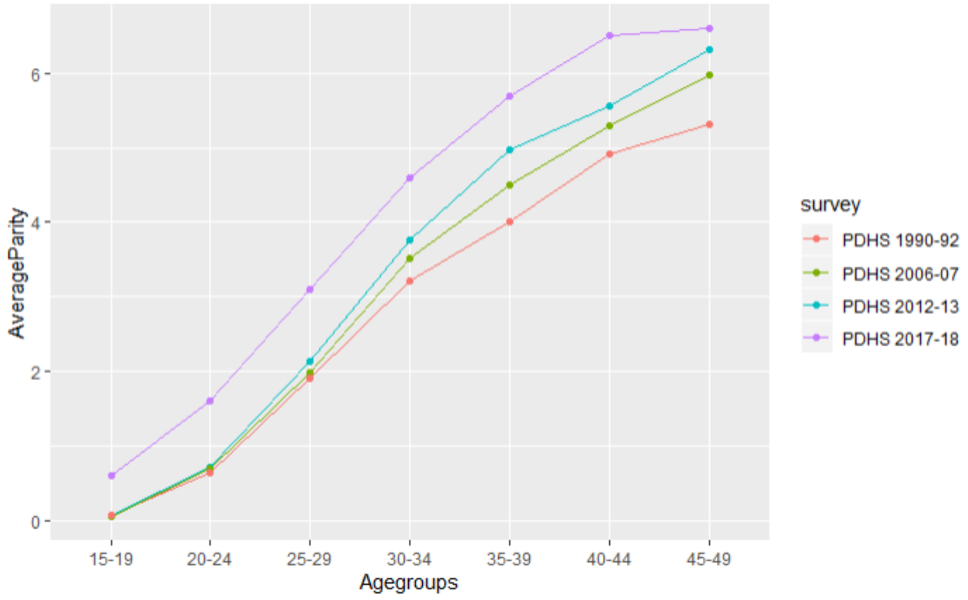

All surveys had consistent trend in observed average parities which is also indication of good quality of data.

2. Assessment of Current Fertility Data

Multiple births in all four PDHS surveys were observed less than 2% so no issue was found in current fertility data.

2.1 Pattern of Age of Respondent (Mother) for Identification of Misreporting and Age Heaping

Brass (1981) emphasized the importance of age in computation of fertility measures. It may result in errors in placement of births and lifetime fertility (parities) in various age groups. Age also plays vital role in calculation of P/F ratio and Relational Gompertz model, so it necessitates examining age of women. In all PDHS, majority of sampled women were between ages 20-34. Maximum number of women interviewed fall in age group 25-29 (Figure4).

Figure 4: Age Distribution of Women by Single Year of Age

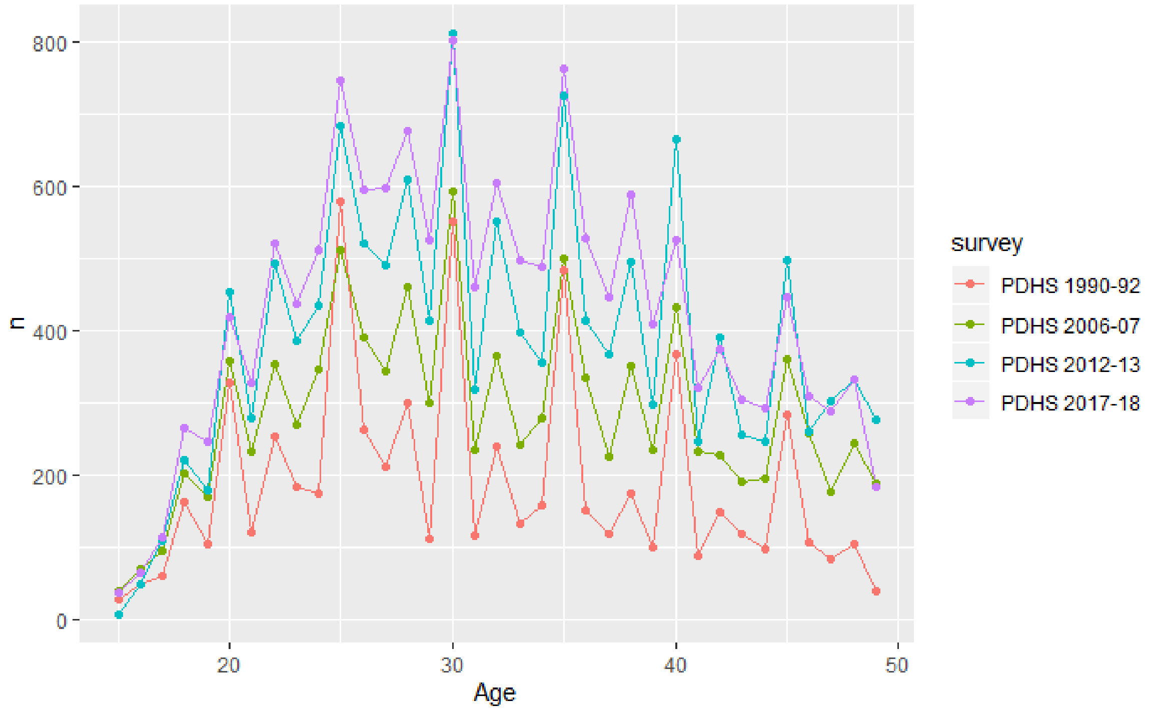

Figure 5: Age Distribution of Women by 5 Year Age Group

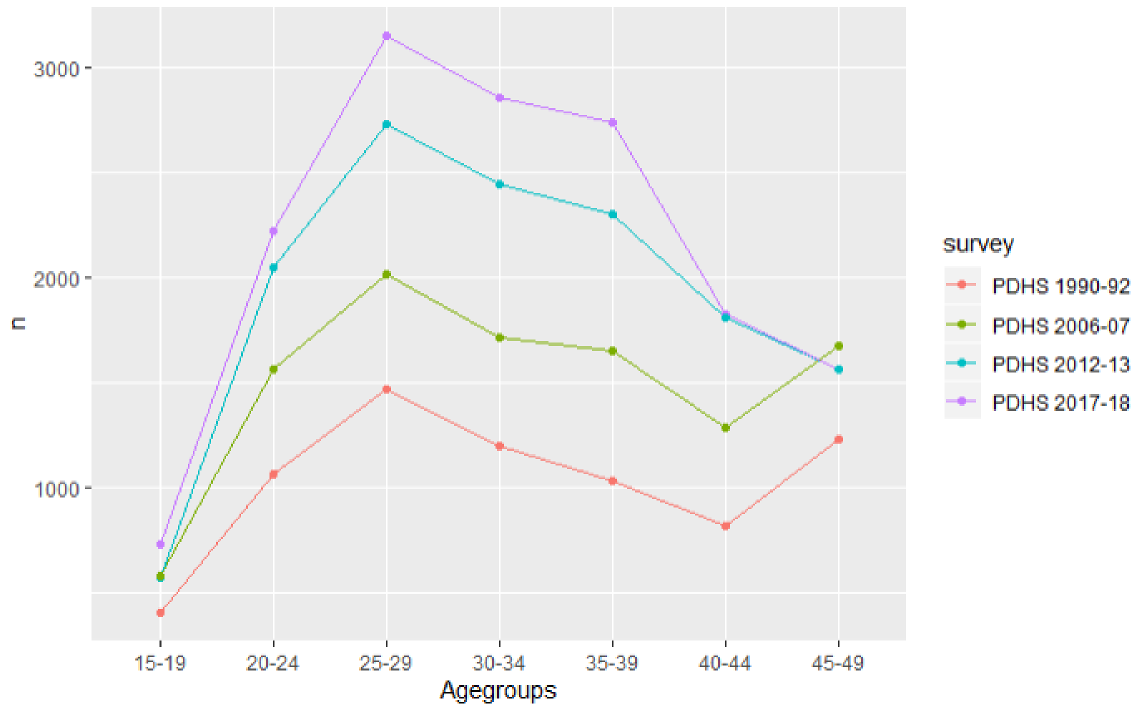

Figure 6: Age Distribution of Child

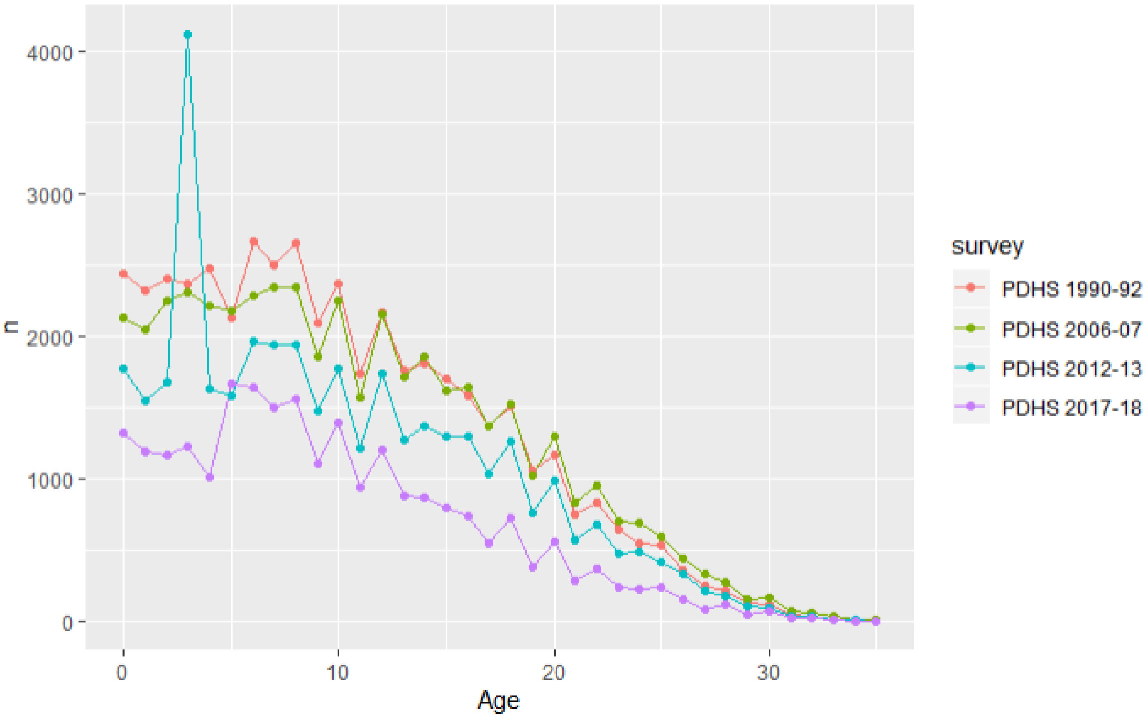

Age heaping is obvious from graph for women age and it can be observed that pattern is same in four PDHS data sets (Figure 4). Graph for age of child is also showing some distortion particularly for births occurring one year preceding the survey (Figure 6). That might be due to errors in reporting date of births of children.

Fertility rates are affected by errors and omissions in reporting age and age heaping. Age distribution of women for the four surveys showed age heaping. It is evident from above graph that there was digit preference. This age heaping may be due to age misreporting or due to enumerator wrong age assessment. To investigate digit preference, table of percentage along with age of women (Table 5) for three surveys is documented as:

Table2: Preference of Digit for Age of Women

Age of Women	PDHS 2017-18	PDHS 2012-13	PDHS 2006-07	PDHS 1990-92
19	1.6%	1.3%	1.7%	1.6%
20	2.8%	3.3%	3.6%	5.0%
24	3.4%	3.2%	3.5%	2.6%
25	5.0%	5.1%	5.1%	8.8%
29	3.5%	3.1%	3.0%	1.7%
30	5.3%	6.0%	5.9%	8.3%
34	3.2%	2.6%	2.8%	2.4%
35	5.1%	5.4%	5.0%	7.3%
39	2.7%	2.2%	2.4%	1.5%
40	3.5%	4.9%	4.3%	5.6%
44	1.9%	1.8%	2.0%	1.5%
45	3.0%	3.7%	3.6%	4.3%

Preference of digit for age ending at 0 and 5 was obvious from above table in all surveys. Preference for digit ending in 0 and 5 were more significant in PDHS 1990-91. Whipple's index was also computed for all surveys for age of women 18 to 47 (Engelhardt, 2005, Al Zalak and Goujen, 2017).

Table3: Preference of Digit and Incompleteness for Age of Women and Age of Child

Survey	Whipple's Index	Myer's Blended Index for Age of Women (15-44)	Myer's Blended Index for Age of Children (0-59 months)	Myer's Blended Index for Age of Children (0-29 years)	Women (%age of Incompleteness)	Women (%age of Incompleteness) Year of Birth or A ge of Women Imputed	Children (%age of Incompleteness)
PDHS 2017-18	129.30%	6.9	2.9	3.4	58%	1.86%	0%
PDHS 2012-13	150.20%	11.4	3.9	3.8	76%	1.57%	0%
PDHS 2006-07	146.60%	10.7	2.7	4.9	74%	0.21%	5.70%
PDHS 1990-91	204.80%	20.9	3.3	6.3	70%	0.14%	3.90%

The value of Whipple's index for both recent surveys lied between 125 and 174.9 so according to Shryock, Siegel and Stockwell (1976), data was classified as rough. For PDHS 1990-91, value of Whipple's index was above 175 so data can be declared as very rough. Myer's Blended Index was a variant of Whipple's index. It showed that quality of data had improved in PDHS 2012-13, PDHS 2017-18 as compared to both past PDHS. We observed that percentage of completeness for age of women was quite low in all surveys so imputation was used for dates. Imputation is problematic

if age or years of birth of women both are not given and both imputed. Incompleteness of information for age of women for all PDHS showed that year or age was given for women so there is no need to worry about imputed dates. For child incompleteness was assessed more strictly and found that 96% and 94% cases had complete information for both year and month. Data quality improved for PDHS 2012-13 and PDHS 2017-18, as 100% complete information about age of child is available. Age misreporting was observed in PDHS 1990-91 and PDHS 2006-07 (Nasir, 2013). Problem of age misreporting was covered in current study as age grouping was used in computation of fertility estimates (Figure 5).

2.2 Omissions and Displacements of Births

This type of error is also quite common in DHS data (Pullum and Becker 2014; Arnold 1990; Pullum 2006; Schoumaker 2011; Al Zalak and Goujon, 2017). It is shifting of birth in one calendar year to other calendar year. An obvious spike was observed for 4 years to 5 years, 5 to 6 years, 6 to 7 years respectively for PDHS 1990-91, PDHS 2006-07, PDHS 2012-13 and 2017-18. After these fall in age is observed in all surveys (Figure7).

Figure7: Births by 9 Year before Survey

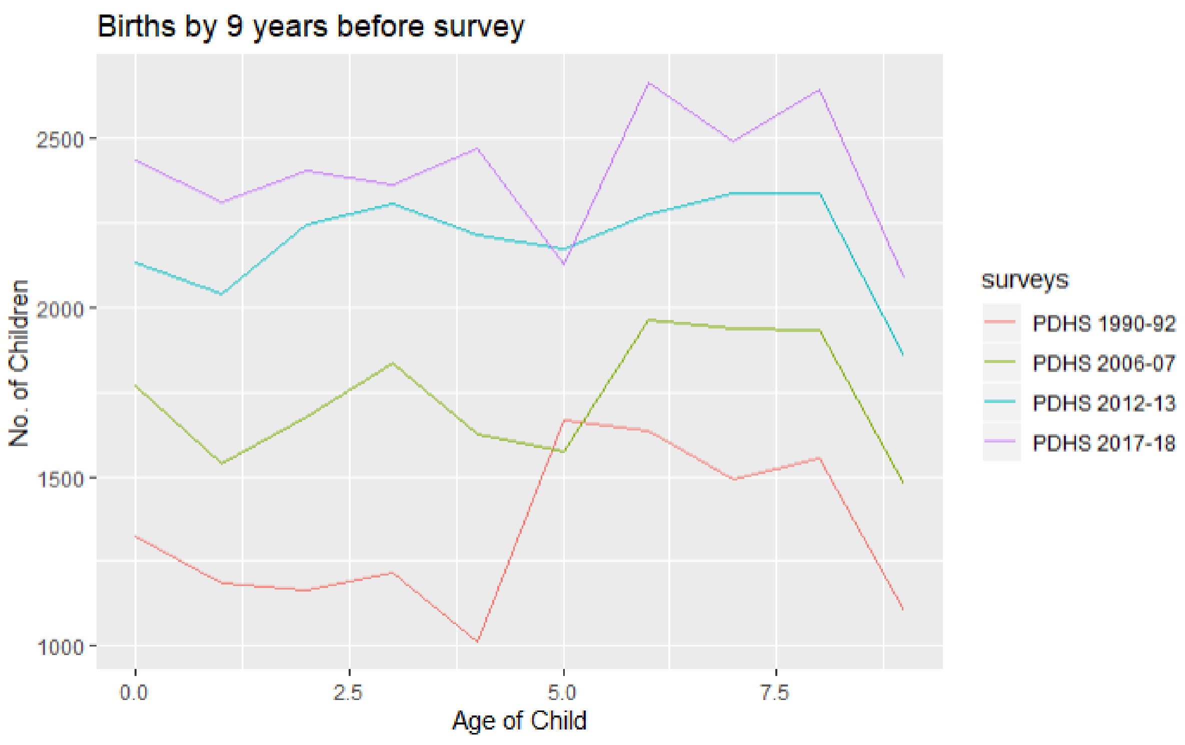

Figure 8: Births by 5 Year before Survey

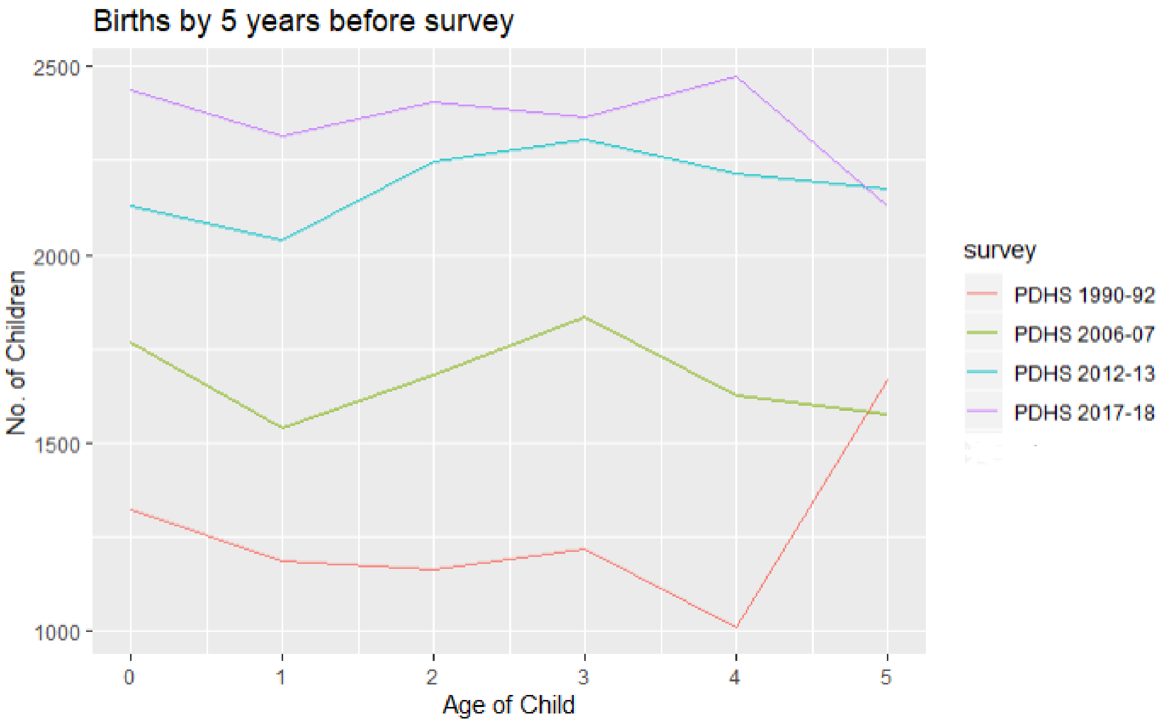

Data quality assessment had shown that the data collected in all PDHS surveys had minor errors. If correctional measures are applied, it will distort the integrity of data (Hlabana, 2006). To fix problem of minor errors and omissions in the fertility data, Relational Gompertz model is recommended technique in the demographic literature.

References

1. Al Zalak, Z., & Goujon, A. (2017). *Assessment of the data quality in Demographic and Health Surveys in Egypt*. Österreich : Vienna Institute of Demography , Austrian Academy of Science.
2. Arnold, F. (1990). Assessment of the Quality of Birth History Data in the Demographic and Health Surveys. An Assessment of DHS-I Data Quality. DHS Methodological Reports No. 1.Columbia, Maryland: Institute for Resource Development / Macro System, Inc.
3. Brass W. (1981). The use of the Relational Gompertz model to estimate fertility. Proceedings of the International Union for the Scientific Study of Population Conference, Manilla, Liege.

4. Engelhardt, H. (2005). *Recent Trends and Components of change in Fertility in Egypt.* Interim Report POP, IIASA, IR-05-024.
5. Hlabana, T. K. (2006). *Application of the P/F Ratio Method in Estimating Fertility Levels in Lesotho* (Doctoral dissertation, University of KwaZulu-Natal, Durban).
6. Nasir, J. A. (2013. *Fertility transition in Pakistan: neglected dimensions and policy implications* (Doctoral Dissertation, University of Southampton).
7. Preston, S.H, Heuveline, P. and Guillot, M. (2001). Demography: Measuring and Modelling Population Processes. Basil Blackwell, London.
8. Pullum, T. (2006). An Assessment of Age and Date Reporting in the DHS Surveys, 1985-2003. DHS Methodological Reports No 5. Calverton, Maryland, USA: Macro International.
9. Pullum, T.W. & Becker, S. (2014). Evidence of Omission and Displacement in DHS Birth Histories. DHS Methodological Reports No. 11. Rockville, Maryland, USA: ICF International.
10. Schoumaker, B. (2011) Omissions of Recent Births in DHS Birth Histories in Sub-Saharan Africa: Measurement and Determinants. Paper presented at the annual meeting of Population Association of America, Washington, D.C., USA. http://paa2011.princeton.edu/papers/112255
11. Shryock, H., Siegel, J. & Stockwell E. G. (1976). The Methods and Materials of Demography, Condensed Edition. New York: Academic Press.
